# Combined Effects of Estrogen and Mechanical Loading on Anterior Cruciate Ligament Fibroblast Biosynthesis

**DOI:** 10.1100/tsw.2005.6

**Published:** 2005-01-14

**Authors:** Xuhui Liu, Zong-Ping Luo

**Affiliations:** Sports Medicine Research Center, Department of Orthopedic Surgery, Baylor College of Medicine, 6550 Fannin Street, Suite 451, Houston, TX 77030, USA

**Keywords:** estrogen, mechanical loading, gene expression, RT-PCR, ACL, fibroblast, collagen

## Abstract

For some time, estrogen has been suspected to play a negative role in anterior cruciate ligament (ACL) fibroblast biosynthesis; however, reports on this issue have been controversial. In a recent study, our group demonstrated a negative combined effect of estrogen and mechanical loading on the gene expression of major extracellular matrix component molecules in ACL fibroblasts.

The anterior cruciate ligament (ACL) is the primary ligament in the knee joint to resist anterior translation and tibial rotation, and is the most vulnerable ligament in sport-related injuries[1,2,3]. Female athletes appear to be four to eight times more likely to sustain ACL injuries than male athletes[4,5,6]. More than 38,000 ACL tears are projected to occur in female varsity intercollegiate and high school athletes each year[7]. Because of their severity, ACL injuries often require surgery to create a stable knee, followed by a dedicated rehabilitation program and adjustment of sport activities.

ACL tissue consists of an extracellular matrix and cells, which are largely fibroblasts. The extracellular matrix of ACL consists of types I and III collagens, proteoglycans, and other minor molecules. The extracellular matrix is generated by the fibroblasts and determines the mechanical properties of ACL. Female reproductive hormones have been considered a major factor contributing to the higher incidence of ACL injuries in females. Since the ACL sustains mechanical loading during movement, mechanical force has also been considered a key factor in this issue. However, the exact mechanism was unknown. In a recent study of combined regulation of estrogen and cyclic tension on ACL fibroblasts biosynthesis[8], our group showed that cyclic tensile loading alone increased the gene expression of collagen I, but did not affect that of collagen III and biglycan. We also found that estrogen alone increased the gene expression of collagen I and III, but not of biglycan. However, combined administration of estrogen and cyclic loading inhibited the gene expression of all three genes. Our results suggested that estrogen and mechanical force might contribute to the higher incidence of female ACL injuries by affecting the mechanical properties of ACL through regulation of fibroblast biosynthesis.

Under a physiological cyclic mechanical loading of 5% strain at 0.5 Hz, 17β-estradiol was administered at three physiological levels mirroring those of the menstrual cycle: $10^{-11}$ *M* (follicular phase), $10^{-10}$ *M* (luteal phase), and $10^{-9}$ *M* (ovulatory phase), and at two therapeutic levels: $10^{-8}$ *M* and $10^{-7}$ *M* using a vehicle of 100% ethanol to fibroblasts derived from porcine ACL. After 24 h, the total RNA of fibroblasts were isolated and the message RNA level of types I and III collagen, as well as another important member of the small leucine-rich proteoglycan family (biglycan), were analyzed using reverse transcript-polymerase chain reaction (RT-PCR). We found that neither cyclic tensile loading nor estrogen downregulated the gene expressions of collagens I and III and biglycan when applied individually. However, combined administration of estrogen and cyclic loading inhibited the gene expression of all three genes.

Though estrogen has been suspected to play a negative role in ACL fibroblast biosynthesis for some time, previous reports on this issue are controversial. Except for Yu et al., who showed dose-dependent decreases in cellular proliferation and type I procollagen synthesis in fibroblasts when exposed to increasing levels of estrogen[9], most other research showed increasing regulation of collagen biosynthesis of estrogen. Slauterbeck et al. reported that fibroblasts derived from rat skin showed increased collagen synthesis of approximately 76%, while those from rat lung showed an increase of approximately 25% after estrogen administration[10]. Estradiol was found to induce a twofold and an eightfold increase in radioactivity of newly synthesized collagen and insoluble collagen in rat uterus cells. Estrogen was also shown to increase mRNA expression of type I collagen in murine osteoblasts[6,11]. Administration of mechanical loading was also found by many researchers to increase collagen types I and III biosynthesis. Yu and his co-workers found that when uniaxial cyclic stretch was applied on ACL cells at 10 cycles/min with 10% length stretch for 24 h, mRNA expressions of the collagen types I and III were increased[12]. Liu et al. found a higher level of type I collagen when the ACL fibroblasts experienced equibiaxial cyclic stretch, although no significant increase in type III collagen was found[13]. In another ACL fibroblast study, Hosokawa et al. found increased collagen synthesis after a 24-h equibiaxial cyclic tensile load[14]. By combining these two factors, we revealed their combined negative regulation on ACL fibroblast biosynthesis for the first time[8].

Our study clarified the contribution of estrogen at the gene expression level to the higher incidence of female ACL injuries. By applying physiological mechanical loading and estrogen on ACL fibroblasts, we found the gene expression of three major extracellular matrix components molecules — collagen types I and III and biglycan — significantly downregulated. This finding was supported by previous clinical and animal studies reporting that estrogen level is related to ACL injuries. In clinical observations during pursuit of various sports[15,16], it has been observed that female athletes suffered ACL injuries up to eight times more frequently than their male counterparts. A higher incidence of ACL injuries in female athletes was noted during the ovulation period when estrogen reached a surge level. Clinical studies have also shown that ACL laxity increases with the increasing level of estrogen[2,3]. In an animal study using ovariectomized rabbits, estrogen administration led to a decrease of the failure load of the ACL[17]. In conclusion, our study discovered a negative combined effect of estrogen and cyclic mechanical loading on major extracellular matrix component molecule gene expression in ACL fibroblast. Hopefully, this will provide new insight into innovative prevention and treatments for female ACL injury.

## BIOSKETCHES

**Xuhui Liu, M.D**., Postdoctoral Associate, Department of Orthopedic Surgery, Baylor College of Medicine, Houston, TX (2001–present). Dr. Liu has designed, organized, and conducted novel research methods in the field of orthopedics, revealing previously unknown biomechanical and biochemical properties of numerous molecules in articular cartilage and ligaments. He has authored four papers on his work, which have been published in major scientific journals, with several more articles currently in preparation. His research interests include molecular and cellular biomechanics, molecular and cellular biology, nanotechnology, articular cartilage, ligaments and tendons. Dr. Liu's research interests focus on the biomechanical and biological basis of musculoskeletal diseases, especially articular cartilage and soft tissue disorders, from a molecular and cellular perspective. Currently, his investigations encompass the area of molecular and cellular biomechanical and biological mechanisms of osteoarthritis, anterior cruciate ligament injuries, and other orthopedic disorders. Dr. Liu received a Travel Award for poster presentation in Arthritis Research Conference, June 26–29, 2003, Keystone, CO.

**Zong-Ping Luo, Ph.D.,** Associate Professor, Department of Orthopedic Surgery, Baylor College of Medicine (2002–present) and Director, Sports Medicine Research Center, Department of Orthopedic Surgery, Baylor College of Medicine (2001–present), specializing in molecular and cellular biomechanics and its applications in sport-related injury treatment and prevention. Over the past 15 years, Dr. Luo has established an international reputation for research on biomechanical applications in the field of orthopedics. He has published over 80 papers in national and international academic journals, and has presented his research findings at numerous scientific conferences in the U.S. and abroad. He holds a Ph.D. in biomechanics from University of California at Berkeley. His research interests include molecular and cellular biomechanics, orthopedic biomechanics, sports medicine, nanotechnology, and soft tissue injuries. Dr. Luo's research interests focus on clinical-related biomechanics of the musculoskeletal system, especially articular cartilage and ligaments of the joints. Currently, investigations have covered the areas of molecular and cellular biomechanical mechanism of osteoarthritis (the most common form of arthritis), anterior cruciate ligament injury, shoulder ligament injury, flat foot, and other orthopedic disorders. Dr. Luo has received The Whitaker Foundation Research Award in Biomedical Engineering (1993), The Arthritis Foundation Research Award in Biomedical Science (1998), and The Arthritis Foundation Research Award in Biomedical Science (2001).
